# A model for predicting the dose to the parotid glands based on their relative overlapping with planning target volumes during helical radiotherapy

**DOI:** 10.1002/acm2.12203

**Published:** 2017-11-06

**Authors:** Cheryl H. Millunchick, Heming Zhen, Gage Redler, Yixiang Liao, Julius V. Turian

**Affiliations:** ^1^ Department of Radiation Oncology Rush University Medical Center 500 South Paulina Street, Ground Floor Atrium Chicago IL USA

**Keywords:** mean parotid dose, prediction model, parotid sparing

## Abstract

The sparing of the parotid glands in the treatment of head and neck cancers is of clinical relevance as high doses to the salivary glands may result in xerostomia. Xerostomia is a major cause of decreased quality of life for head and neck patients. This paper explores the relationship between the overlap of the target volumes and their expansions with the parotid glands for helical delivery plans and their ability to be spared. Various overlapping volumes were examined, and an overlap with a high statistical relevance was found. A model that predicts exceeding tolerance parotid mean dose based on its fractional overlapping volume with PTVs was developed. A fractional overlapping volume of 0.083 between the parotid gland and the high dose PTV plus 5 mm expansion – was determined to be the threshold value to predict parotid D_mean_ > 26 Gy for parotids that overlap with the high dose PTV plus 5 mm expansion. If the parotid gland only overlaps with the intermediate dose target (and/or low dose target) and the overlapping volume of the parotid gland and the intermediate dose target is less than 25%, the parotid mean dose is likely less than 26 Gy. If the parotid overlaps with the low dose target only then the mean dose to the parotid is likely to be less than 26 Gy. This finding will prove as a very useful guide for the physicians and planners involved in the planning process to know prior whether the parotid glands will be able to be spared with the current set of target volumes or if revisions are necessary. This work will serve as a helpful guide in the planning process of head and neck target cases.

## 1 INTRODUCTION

The treatment of head and neck cancers has evolved in recent years from static IMRT (generally 7–9 fields) to the state of the art arc‐based technologies of Volumetric Modulated Arc Therapy (VMAT) and helical delivery i.e. Tomotherapy, which has been necessary as a result of the complexity of the target volumes and their proximity to numerous organs‐at‐risk (OARs). OARs of concern are: the spinal canal, submandibular glands, brachial plexi, mandible, cochleae, optic apparatus, superior/inferior constrictors, and the parotid glands. The sparing of the parotid glands is the main focus of this paper. This is of clinical importance since high doses to the salivary glands results in a reduction of salivary output and a change in its composition, which in turn may lead to xerostomia, a major cause of decreased quality of life for this set of patients.^1^, ^2^ Per Roesink et al, treatment planners should aim for a mean parotid gland dose less than 39 Gy leading to a complication probability of 50%.^3^ However, per studies by Eisbruch et al, in order to retain salivary function of the parotid glands, the mean parotid gland dose must be at or below 26 Gy.^4^ A correlation between mean parotid dose and the fractional reduction of stimulated saliva output at 6 months after the completion of radiation therapy was observed in studies of Chao et al^5^ In recent studies by Gensheimer et al, the overlap of the parotid gland with a 1 cm expansion of the combined targets (combining the various dose levels of the targets) was found to be the best predictor for the mean dose of the parotid glands (D_mean_) for static IMRT cases.^6^ Prior studies by Hunt et al concluded that dosimetric sparing of the parotid glands resulting in a Dmean < 26.1 Gy for static IMRT plans is feasible if the parotid‐PTV overlap is less than approximately 20%.^7^ The focus of this study is to determine which parameter is capable of accurately predicting the mean dose of the parotid glands for plans developed using helical arc available on Accuray's Tomotherapy System (Accuray Inc, Sunnyvale, CA, USA). As discussed by Gensheimer, these findings will allow the clinical staff working on the case to determine if the target volumes require alteration to achieve dosimetric objectives before a considerable amount of time is spent planning the case only to discover that the desired sparing of the parotid glands is unachievable. This could avoid delaying the start date of the patient and utilize limited resources more efficiently.

## 2 METHODS AND MATERIALS

### 2.A Selection criteria

The following criteria were applied for inclusion of head and neck patients in this study: intact bilateral parotid glands and 2–4 simultaneously treated targets with prescriptions ranging from 54 Gy to 70 Gy. The dose schemes and the primary disease sites for the patients in this study are characterized in Table 1 and Table 2. Both parotid glands were contoured with the inclusion of deep and superficial lobes of the glands. The range of the parotid gland volumes was from 11.35 cc to 55.18 cc, and the mean parotid gland volume was 29.90 cc. Efforts were made to spare the parotid glands while ensuring that the target(s) coverage was not significantly compromised. The criterion used for determining if the target had acceptable coverage was 90% coverage of the target by the prescription dose and that the compromised target coverage was not overlapping with the GTV. Plans with unacceptable PTV coverage (V100% < 90% for PTV with the highest dose level) were excluded from the analysis. After applying all the inclusion criteria, 37 clinical plans were included in this study. Treatment planning was performed using Tomotherapy software version 5.1 utilizing the 2.5 cm field size (fixed jaws), 0.215 couch pitch, 3.5 modulation factor and fine dose resolution settings (256 × 256 × #slices).

**Table 1:** Disease sites

Primary site	Number of cases per site
Base of tongue	9
Floor of mouth	2
Hypopharynx	3
Larynx	9
Lip	1
Nasopharynx	2
Oral cavity	2
Oral tongue	1
Oropharynx	2
Parotid gland	1
Thyroid	1
Tonsil	9
Unknown primary	1

**Table 2:** Prescription levels

Dose levels (Gy)	Number of cases per dose level
70, 66, 59.4, and 56	2
69.96, 66, 59.4, and 54.45	1
70, 63, and 56	5
70, 59.4, and 56	14
69.96, 59.4, and 54.45	5
66, 59.4, and 56	2
66, 60, and 54.45	8
69.96 and 59.4	1
66 and 54	2
60 and 54	3

### 2.B Parotid dose vs. overlap with combined PTVs and expansions

The mean parotid dose was first analyzed against the relative overlapping volume with the combined PTV (union of PTVs for all dose levels) and its expansions. The following three parameters were computed: fractional overlap of the parotid glands with the combined targets (OLV_CT_), fractional overlap of the parotid glands with the 0.5 cm isotropic expansion of combined PTV (OLV_CT05_), and fractional overlap of the parotid glands with the 1 cm isotropic expansion of the combined PTV (OLV_CT10_). Throughout this article, the term “fractional overlap” is defined as the ratio of the overlapping volume between the parotid and the target contour to the volume of the parotid contour. Univariate analysis was done using linear regression between parotid D_mean_ and each of the three overlapping parameters. Multivariate analysis was done using a linear regression model with all three overlapping parameters, followed by a stepwise model selection to determine the combination of these overlapping parameters that best predicts the mean parotid dose. The backward stepwise regression is done with an automatic algorithm that starts with a full model, and in each step subtracts a variable based on the Akaike Information Criterion (AIC), until the best model is achieved. Receiver Operating Characteristic (ROC) analysis was also performed to compare the predictive power of different overlapping volumes. Each parotid dose evaluated is considered “positive” if the mean dose is greater than 26 Gy, and “negative” if the mean dose is equal or less than 26 Gy.

### 2.C Parotid dose vs. overlap with PTVs of various dose levels

The plans were further stratified by noting whether the parotid volume overlaps with PTVs at various dose levels—high dose: PTV70 or PTV66, medium dose: PTV63 or PTV60, and low dose: PTV56 or PTV54. Here “PTVx” denotes the PTV that is prescribed to receive x Gy.

**Figure 1:**
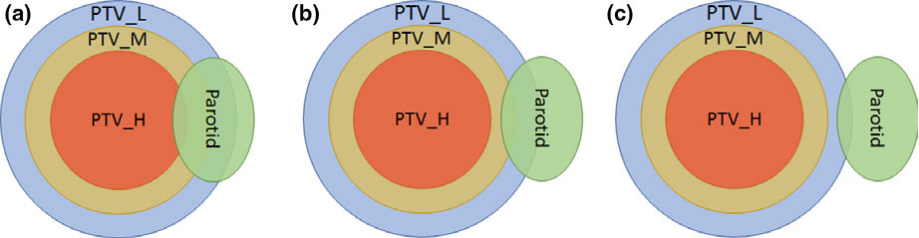
Three scenarios of parotid overlapping with PTV: (a) Parotid overlaps with high dose PTV (PTV_H), (b) Parotid does not overlap with PTV_H, but overlaps with medium dose PTV (PTV_M), and (c) Parotid does not overlap with PTV_H or PTV_M, but overlaps with low dose PTV (PTV_L).

Specifically, plans were stratified into three groups: (a). Parotid overlaps with high dose PTV (b). Parotid does not overlap with high dose PTV, but overlaps with medium dose PTV (c). Parotid does not overlap with high or medium dose PTV, but overlaps with low dose PTV, as shown in Fig. 1.

### 2.D Parotid dose vs. overlap with high dose PTV and its expansion

Initial observation of these three groups stratified by dose levels that are listed above shows that no plan in group 3 exceeds the parotid D_mean_ tolerance of 26 Gy. Data in group 2 show a clear cutoff of relative overlapping volume of around 20%. Data in group 3 show a general trend of higher parotid D_mean_ with higher overlapping volume. However, no clear cutoff value was observed. Further analysis was performed to determine the relation between parotid D_mean_ and parotid overlap with high dose PTV (OLV_HD_), as well as with 0.5 cm expansion of the high dose target (OLV_HD05_).

Linear regression was done to examine the correlation between parotid mean dose and either OLV_HD_, or OLV_HD05_. ROC analysis was then performed to compare these two parameters as parotid dose predictors and identify optimal thresholds. ROC curves were then generated against OLV_HD_ and OLV_HD05_. The areas under the ROC curves were compared to select the best predictor. After selecting the best predictor, an optimal point on the ROC curve was identified to establish a threshold value.

The statistical analysis in this study was performed using R version 3.2.3. (R Foundation for Statistical Computing, www.cran.r-project.org).

## 3 RESULTS

### 3.A Parotid dose vs. overlap with combined PTV and expansions

**Figure 2:**
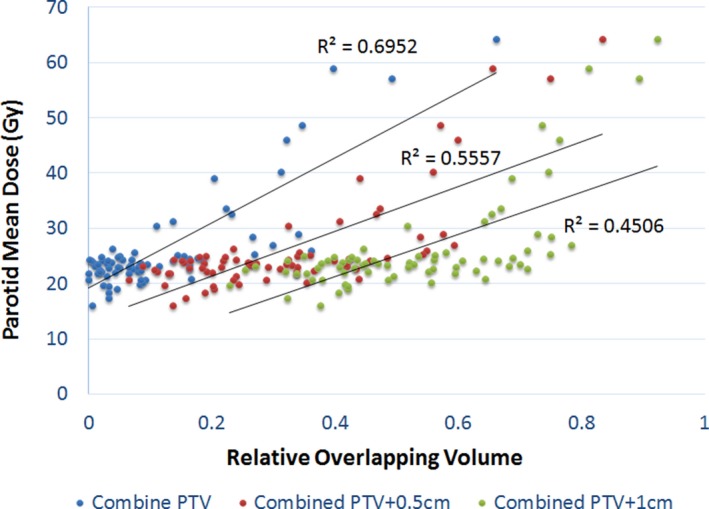
Linear regression of parotid mean dose vs. fractional overlapping with combined PTV and its expansions.

**Figure 3:**
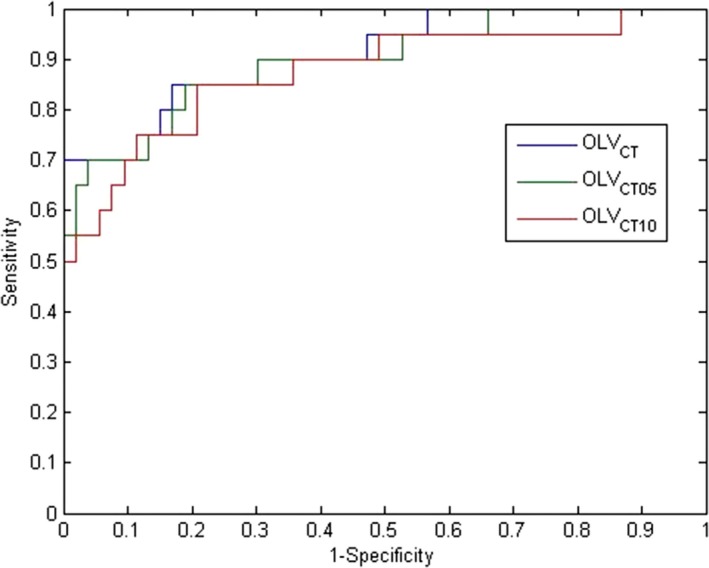
ROC analysis of combined target volume and its expansions as predictors for parotid D_mean_ > 26 Gy.

The mean parotid dose vs. OLV_CT_, OLV_CT05_, and OLV_CT10_ are depicted in Fig. 2. Linear regression resulted in R^2^ values of 0.6952, 0.5557, and 0.4506, with corresponding *P*‐values of < 0.01, < 0.01, and 0.36, respectively. The backward stepwise model selection also reduced the model to a single independent variable—OLV_CT_. The ROC curves for the three volume predictors are shown in Fig. 3, with parotid D_mean_ > 26 Gy as “positive”. The Area Under the Curve (AUC) were found to be 0.9075, 0.8972, and 0.8755 for OLV_CT_, OLV_CT05_, and OLV_CT10_, respectively. The above results show consistently that OLV_CT_ is the best predictor of parotid D_mean_ among the three.

### 3.B Parotid dose vs. overlap with PTVs of various dose levels

**Figure 4:**
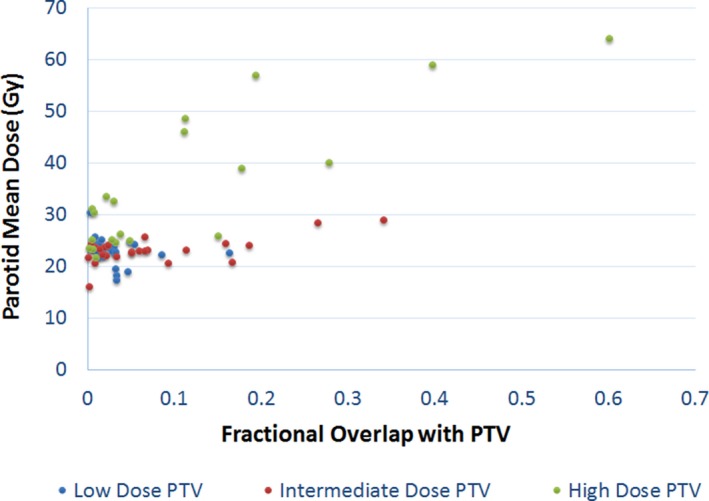
Parotid D_mean_ vs. fractional overlap volume between parotid and PTVs with various dose levels. (High Dose: 66–70 Gy, Intermediate dose: 60–63 Gy, Low dose: 56–60 Gy).

Figure 4 shows the parotid mean dose vs. its fractional overlapping volume with PTVs at various dose levels. It is shown that:

(a) When the parotid is only overlapping with the low dose PTV (56–60 Gy), the parotid mean dose is lower than 26 Gy. The maximum overlapping fractional volume in our dataset was 0.16.
(b) When the parotid is overlapping with the intermediate dose PTV but not the high dose PTV, 23 of 25 data points have parotid mean dose less than 26 Gy, all with fractional volume overlap less than 0.2. There were two cases where the parotid mean dose exceeded 26 Gy and the fractional volume overlap was 0.26 and 0.34, respectively. Our data seem to suggest a cutoff value, for fractional volume, between 0.2 and 0.25, however, there is no statistical significance associated with it due to lack of positive cases.
(c) When the parotid is overlapping with the high dose PTV, there were multiple positive cases where the parotid mean dose exceeded 26 Gy. A general trend of higher parotid mean dose with higher fractional volume overlap is observed, which makes intuitive sense. However, there is a rather large variation in the data.

### 3.C Parotid dose vs. overlap with high dose PTV and its expansion

**Figure 5:**
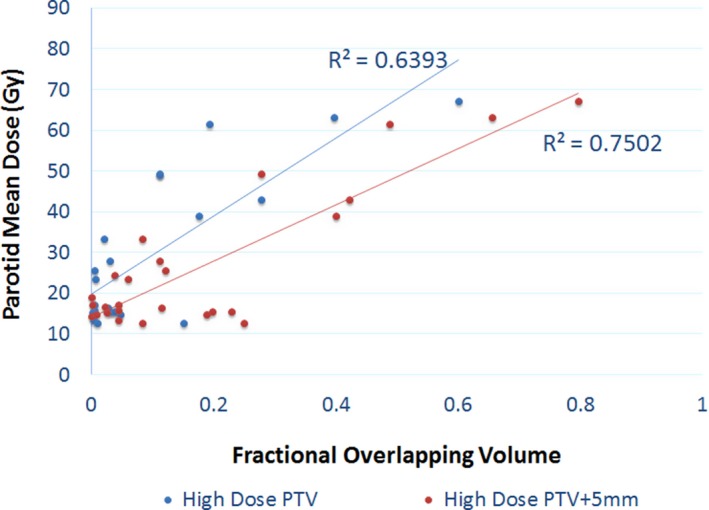
Parotid mean dose vs. fractional overlap between parotid and high dose PTV and its 5 mm expansion volume.

Parotid mean dose vs. OLV_HD_ and OLV_HD05_ are plotted in Fig. 5. Linear regression resulted in R^2^ values of 0.70 for OLV_HD_ and 0.82 for OLV_HD05_, both with associated *P*‐values < 0.01. OLV_HD05_ seems to be better correlated with parotid mean dose.

**Figure 6:**
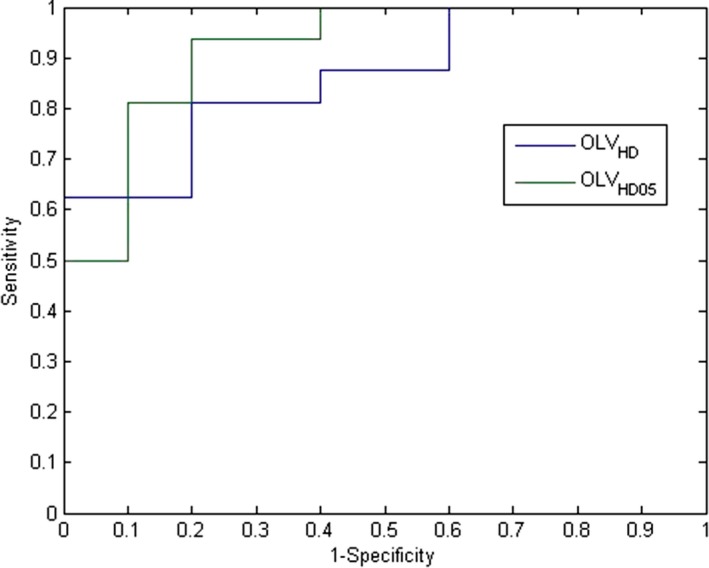
ROC analysis of OLVHD, OLVHD05 as predictors for parotid D_mean_ > 26 Gy.

ROC analysis was performed for OLV_HD_ and OLV_HD05_. The corresponding ROC curves are shown in Fig. 6. The AUCs are found to be 0.8625 and 0.9188, respectively. Again, OLV_HD05_ seems to be the better predictor. OLV_HD05_ also has a higher AUC than OLV_CT_, and therefore was determined to be the best parotid D_mean_ predictor. The optimal point with best overall sensitivity and specificity was identified with a threshold value of 0.0829, a sensitivity of 0.81, and a specificity of 0.90. This demonstrates that an OLV_HD05_ of greater than 0.0829 (or 8.29%) is a reasonable threshold for predicting a mean parotid D_mean_ greater than 26 Gy.

## 4 DISCUSSION

It is interesting to observe that when analyzing combined targets and their expansions, the overlap with PTVs only is the better fit compared to overlapping with PTV expansions. This differs from the findings of Gensheimer et al.^6^ However, it is plausible, considering Tomotherapy's ability to produce very steep dose gradients, that partial overlap with low and even intermediate dose targets may not be a critical issue for parotid mean dose sparing.

This hypothesis was indirectly proved by the analysis after stratifying the data based on different dose levels. When the parotid is partially overlapping with the low dose target only, we were always able to meet the parotid mean dose criteria, and no clear trend can be observed from the scatter plot. When the parotid overlaps with an intermediate dose target, there seems to be a clear cutoff at 0.2–0.25, but with no statistical significance due to lack of “positive” data.

Overlapping with high dose data was the most powerful predictor. It's also interesting to see that overlapping with 5 mm PTV expansion was an even better predictor. This differs from the combined PTV result, but agrees with Gensheimer et al. with respect to an expansion which may be a result of the effective field size of 0.5 cm of the Tomotherapy plans.

These findings are intended to be beneficial to treatment planners as the planner can increase efficiency by anticipating whether the desired parotid sparing is achievable prior to planning rather than retrospectively making this discovery after exhausting all potential approaches to achieve the desired goal of sparing the parotid glands. The cutoff value of 8.3% for the overlap between the parotid glands and the 0.5 cm expansion of the high dose targets can be an extremely useful tool for the planner. If the PTVs provided by the physicians yield an OLV_HD05_ equal to or greater than 8.3%, then the planner should bring this to the attention of the physician and discuss whether the physician should modify the PTVs to achieve parotid sparing or keep the PTVs as is but accept the possibility of not being able to spare the intended parotid gland(s). This allows for a much more efficient use of resources in the planning process.

This model intentionally does not differentiate between the contralateral and ipsilateral parotids as the model is purely based on overlap volume. It is reasonable to assume that the ipsilateral parotid will tend to share more of an overlap with the target especially the high dose target which in turn will yield an unfavorable mean dose prediction with this model.

The Tomotherapy parameters that were used in this study—2.5 cm field size, .215 pitch, and 3.5 modulation factor—is the standard of practice at our clinical site. These parameters yield plans with desirable dose distributions while maintain reasonable treatment times. Reasonable treatment times are essential not only for efficiency but also for delivery accuracy. It is feasible that a smaller field size and pitch and/or larger modulation factor can result in better parotid sparing but may also result in substandard delivery accuracy and prolonged treatment time. It is beyond the scope of our paper to prove or disprove this hypothesis.

The separation between the ROC curves are not large mainly due to the similar nature of the quantities—overlap between the targets and the parotid. Specifically, for the combined targets with various expansions, the ROC curves are very similar indicating a similar performance if chosen as the predictor. As for the high dose target only, the ROC curve for the optimal threshold for OLV_HD05_ is better than any threshold with OLV_HD_ by at least 10% sensitivity and specificity. More significant differences may be observed with a much larger dataset.

The ROC curves for combined PTVs with various expansions (method 1) and PTVs stratified by dose levels (method 2) are not significantly separated within each method. However, these ROC curves cannot be compared directly. The ROC curves in Fig. 3 (method 1) are based on the full data set, while the ROC curves in Fig. 6 (method 2) only includes the data where the high dose target/expansion overlaps with the parotid. When the parotid does not overlap with the high dose target, method 2 essentially yields a perfect prediction (100% sensitivity and specificity). When there is overlap, method 2 yields a slightly higher AUC compared to method 1. Therefore, the overall performance of method 2 is better than method 1.

Future work includes collecting more data to strengthen the presented model, and applying similar analysis to other normal tissues, such as the duodenum in pancreatic cases. Additionally, this analysis can be used for treatment modality comparison, such as Tomotherapy vs. VMAT which is now widely used for the conformal treatment of complicated tumors.

## 5 CONCLUSION

We analyzed the relation between parotid mean dose and the overlapping volume between parotid and various PTVs and their expansions, and found out that:

1. When the parotid is overlapping with the low dose target only (with overlapping volumes all less than 16% in our data set), the parotid mean dose is likely under 26 Gy.
2. When the parotid is overlapping with the intermediate dose target only, but the overlapping volume is less than 25%, the parotid mean dose is likely less than 26 Gy.
3. When the parotid is overlapping with the high dose PTV plus 5 mm expansion, then an overlapping volume of 8.3% could be used as a threshold to predict parotid D_mean_ > 26 Gy.

## CONFLICT OF INTEREST

The authors of this manuscript bear no conflicts of interest.
